# A Novel Chromosomal Translocation Identified due to Complex Genetic Instability in iPSC Generated for Choroideremia

**DOI:** 10.3390/cells8091068

**Published:** 2019-09-11

**Authors:** Nejla Erkilic, Vincent Gatinois, Simona Torriano, Pauline Bouret, Carla Sanjurjo-Soriano, Valerie De Luca, Krishna Damodar, Nicolas Cereso, Jacques Puechberty, Rocio Sanchez-Alcudia, Christian P. Hamel, Carmen Ayuso, Isabelle Meunier, Franck Pellestor, Vasiliki Kalatzis

**Affiliations:** 1 Inserm U1051, Institute for Neurosciences of Montpellier, 34091 Montpellier, France; 2 University of Montpellier, 34095 Montpellier, France; 3 Chromosomal Genetics Unit, Chromostem Platform, CHU, 34090 Montpellier, France; 4 Service of Clinical Genetics, Department of Medical Genetics, Rare Diseases and Personalized Medicine, CHU, 34090 Montpellier, France; 5 Department of Genetics, Institute for Sanitary Investigation, Foundation Jimenez Diaz, 28040 Madrid, Spain; 6 Centre for Biomedical Network Research on Rare Diseases (CIBERER), 28029 Madrid, Spain; 7 National Reference Centre for Inherited Sensory Diseases, CHU, 34295 Montpellier, France

**Keywords:** induced pluripotent stem cells, genetic instability, retinal differentiation, inherited retinal dystrophies, choroideremia, underprenylated cell phenotype

## Abstract

Induced pluripotent stem cells (iPSCs) have revolutionized the study of human diseases as they can renew indefinitely, undergo multi-lineage differentiation, and generate disease-specific models. However, the difficulty of working with iPSCs is that they are prone to genetic instability. Furthermore, genetically unstable iPSCs are often discarded, as they can have unforeseen consequences on pathophysiological or therapeutic read-outs. We generated iPSCs from two brothers of a previously unstudied family affected with the inherited retinal dystrophy choroideremia. We detected complex rearrangements involving chromosomes 12, 20 and/or 5 in the generated iPSCs. Suspecting an underlying chromosomal aberration, we performed karyotype analysis of the original fibroblasts, and of blood cells from additional family members. We identified a novel chromosomal translocation t(12;20)(q24.3;q11.2) segregating in this family. We determined that the translocation was balanced and did not impact subsequent retinal differentiation. We show for the first time that an undetected genetic instability in somatic cells can breed further instability upon reprogramming. Therefore, the detection of chromosomal aberrations in iPSCs should not be disregarded, as they may reveal rearrangements segregating in families. Furthermore, as such rearrangements are often associated with reproductive failure or birth defects, this in turn has important consequences for genetic counseling of family members.

## 1. Background

Cell culture is the backbone of biomedical research, but most human cell lines are genetically modified to drive immortal growth and carry genetic artifacts [1]. Conversely, primary cultures are more genetically faithful but can only be cultured for a limited time prior to de-differentiation or senescence. Pluripotent stem cells (PSCs) have revolutionized this field as they can self-renew indefinitely and undergo multi-lineage differentiation [2]. A main source of human PSCs is embryonic stem cells, derived from the inner cell mass of the blastocyst. However, the associated ethical considerations hinder their availability and application due to strict regulations. The advent of induced pluripotent stem cell (iPSC) technology, which derives from adult somatic cells, most commonly fibroblasts [3,4] or peripheral blood circulating monocytes [5], has circumvented these limitations to create a multifaceted tool for human studies [6]. Furthermore, as iPSCs can be generated from the cells of patients with inherited disorders [7], this has opened up a host of possibilities concerning the use of iPSC-derived tissues for disease modelling and therapeutic studies.

One domain that has highly benefited from disease-specific iPSC technology is the study of inherited retinal dystrophies (IRD) [8]. IRDs are characterized by progressive visual impairment due to degeneration of the light-sensing photoreceptors of the neuroretina and the underlying support tissue, the retinal pigment epithelium (RPE). The potential of iPSCs to recapitulate eye development in vitro by differentiating into a stratified neuroretina containing photoreceptors [9,10] and fully functional iPSC-derived RPE [11,12] has resulted in the modelling of an array of IRDs as well as efficiency testing of novel therapies [13,14,15,16,17,18,19,20]. This approach has been particularly pertinent for the IRD choroideremia (CHM; MIM: 303100). CHM is due to mutations in the X-linked gene *CHM*, which encodes Rab escort protein-1 or REP1 [21]. REP1 acts as a chaperon for Rab guanosine triphosphatases (Rab GTPases) to enable their prenylation and delivery to their target membranes [22]. Thus, in the absence of REP1, unprenylated Rab GTPases accumulate in the cytosol. There is a paucity of appropriate disease models to study CHM as both the knockout zebrafish [23] and mouse [24] models are lethal, and the conditional knockout mouse model does not reproduce the disease observed in human males [25]. We thus generated human CHM-specific iPSC-derived RPE and were the first to show that it reproduced the biochemical defect of patients and was an excellent tool for assessing the efficiency of a gene replacement strategy [13,19]. Recently, another team exploited the same approach [26], thus reinforcing its pertinence.

For all their advantages, the disadvantage of using iPSCs is their highly documented genetic instability [27], which can have unforeseen consequences on pathophysiological or therapeutic read-outs. Numerous studies have highlighted the link between chromosomal mis-segregation, replication stress and genomic instability [28]. A pertinent fundamental and clinical question is the impact of chromosomal abnormalities and structural variations on the genomic instability of iPSC lines. In relation to this topic, karyotype analysis of iPSCs generated from two brothers with a typical CHM clinical phenotype detected an uncommonly high frequency of complex rearrangements involving chromosomes 12, 20 and/or 5. We suspected an underlying chromosomal aberration and karyotyped the original fibroblasts from the two brothers, as well as blood cells from all family members. We identified a novel chromosomal translocation denoted t(12;20)(q24.3;q11.2) that segregated from the father to three of the four siblings. We determined that this translocation is balanced and does not impact subsequent retinal differentiation. Importantly, we show for the first time that a chromosomal rearrangement in somatic cells can breed a high level of genetic instability following reprogramming. Therefore, unstable iPSCs should not be automatically discarded, as they can uncover chromosomal aberrations segregating in unsuspecting families. Furthermore, due to the high rate of association of such rearrangements with reproductive failure or birth defects, this in turn has important consequences for future family planning.

## 2. Materials and Methods

### 2.1. Genetic and Clinical Investigations

Haplotype analysis was performed using a polymorphic marker (AR) in the androgen receptor gene, the microsatellite markers DXS8076 (UCSC Human Genome Browser (GRCh37/hg19) Xq21.1:82779309-82779564) and DXS1002 (Xq21.2:85527058-85527406) flanking the *CHM* gene, and an intragenic STR in intron 14 (I-14) of *CHM*, as described [29]. The patients were examined using standard ophthalmologic tests (i.e., refraction, visual acuity, slit-lamp examination and applanation tonometry). Best-corrected visual acuity, kinetic visual fields and full-field electroretinography were performed as previously described [19]. Color fundus images were performed using a Topcon Imagenet (Ophthalmic Imaging Systems, Japan) or a Nidek non-mydriatic automated fundus camera AFC 330 (Nidek Inc., Japan). Fundus autofluorescence and Spectral Domain Optical Coherence Tomography (SD-OCT) imaging were performed with a Combined Heidelberg Retina Angiograph + Spectralis OCT device (Heidelberg Engineering, Dossenheim, Germany).

### 2.2. Skin Biopsy and Fibroblast Culture

The skin biopsies of the proband CHM4 and his brother CHM5 were performed following informed consent at the National Reference Centre for Inherited Sensory Diseases (CHU Montpellier) under the biomedical research authorization number 2014-A00549-38. The explants and emerging fibroblasts were cultured as previously described [29].

### 2.3. Reprogramming of iPSCs

Fibroblasts were reprogrammed using the CytoTune-iPS 1.0 Reprogramming kit (Life Technologies, ThermoFisher Scientific) containing four Sendai virus-based reprogramming vectors expressing *OCT4*, *SOX2*, *KLF4* and *c-MYC* at a multiplicity of infection of 3 for each vector, as previously described (20). Briefly, fibroblasts were seeded on day (D) -1, transduced on D0, and passaged onto feeder cells [13] at D5. Resulting iPSCs were mechanically passaged in ES media (Knockout DMEM containing 20% KnockOut Serum Replacement, 200 mM GlutaMAX, 1% non-essential amino acids, 0.1% β-mercaptoethanol and 1% penicillin-streptomycin; Gibco, ThermoFisher Scientific, Villebon sur Yvette, France) in the presence of 10 µM StemMACS Y-27632 (Miltenyi Biotech, Paris, France). The iPSC clone 2 from CHM5 was subsequently adapted to feeder-free culture conditions on 1:100 dilution of Corning Matrigel hESC-Qualified Matrix (Dominique Dutscher, Brumath, France) in Essential 8 (E8) media (Gibco) and subsequent passages were performed using Versene solution (Gibco).

### 2.4. In Vitro Differentiation Assay

On D0, the iPSCs were dissociated with Accutase (Stemcell Technologies, Grenoble, France) and seeded on ultra-low attachment dishes in E8 medium containing 10 µM Y-27632. At D3, the medium was changed to DMEM/F12 containing 20% KnockOut Serum Replacement, 1% penicillin-streptomycin, 1% GlutaMax, 55mM β-mercaptoethanol and 1% non-essential amino acids. At D7, the differentiated embryoid bodies were seeded onto Matrigel-coated Nunc LabTek chamber slides (ThermoFisher Scientific) and cultured until D17.

### 2.5. Differentiation of iPSC-Derived RPE

The spontaneous differentiation of the CHM5 iPSCs into RPE was performed as previously described [19]. Briefly, iPSCs were grown to confluence and the E8 media was changed to ES media. Pigmented foci were manually dissected, dissociated with 0.25% trypsin, passed through a 40-µm filter and seeded on a 1:30 dilution of Matrigel. The iPSC-derived RPE was used at cell culture passage (P) 3 for all experiments.

### 2.6. qPCR Analysis

RNA was isolated using the QiaShredder and RNeasy mini kits (Qiagen), treated with RNase-Free DNase 1 (Qiagen), and 0.5 µg was reverse transcribed using the Superscript III Reverse Transcriptase kit (Life Technologies). For the quantitative PCR (qPCR) studies, primer sequences were previously reported as follows: endogenous *NANOG*, *SOX2*, *OCT4, LIN28A* and *CHM* [13]; *PAX6*, *ZO1*, *MERTK*, *TYR, RLBP1*, *RDH5* and *BEST1* [19]. RNA from wild type iPSCs [30] or iPSC-derived RPE [19] was used as a positive control, and from fibroblasts as a negative control. Reactions were performed in triplicate using the LightCycler^®^ 480 SYBR Green I Master mix on a LightCycler^®^ 480 II thermal cycler (Roche) and analyzed as described [19]. Quantification was performed using the ΔΔCt method and expression levels were normalized to *GAPDH* expression.

### 2.7. Western Blot Analysis

Cells were collected, resuspended in 2x Laemmli’s sample buffer (Biorad, Marne La Coquette, France), loaded onto an AnyKD precast MiniProtean TGX Stain Free gel (Biorad) and electrotransferred using a Trans-Blot^®^ Turbo™ PVDF Transfer Pack and System (Biorad). Western blot analysis was performed using a monoclonal mouse anti-REP1 antibody (clone 2F1; Millipore, Saint Quentin en Yvelines, France), and a monoclonal mouse anti-β-actin antibody as a loading control, as previously described without modification [19].

### 2.8. Karyotype Analysis

Following informed consent, whole heparinized blood was added to a 25 cm^2^ flask containing Chromosome Medium P (Euroclone S.p.A., Pero, Italy) and incubated at 37 °C under 5% CO_2_ for 48 h. The cells were then sequentially incubated with synchronization solution A (Euroclone S.p.A., Pero, Italy) overnight, solution B for 5 h and colchicine (Sigma-Aldrich, Saint-Quentin Fallavier, France) for 1 h. Fibroblasts and iPSCs were grown to 50% confluence in 75 cm^2^ flasks and 8 cm^2^ dishes, respectively, and processed as previously described [19]. Karyotype analysis was performed on at least 20 metaphases using standard RHG banding procedures. Karyotypes were analyzed using Ikaros software (MetaSystems, Altlussheim, Germany). Multicolor fluorescence in situ hybridization (FISH) labeling [31] was performed with commercial whole chromosome probe kit 24XCyte (Metasystems Probes, Altlussheim, Germany) according to the suppliers’ instructions.

### 2.9. Copy Number Variant Analysis

Genomic DNA was isolated from the fibroblasts of patients CHM4 and CHM5 using the DNeasy Blood & Tissue Kit (Qiagen, Les Ulis, France) according to the manufacturer’s instructions. Array Comparative Genomic Hybridization (aCGH) was performed using SurePrint G3 Human CGH Microarray 60K (Agilent, Santa Clara, CA, USA), according to the supplier’s instructions. Results were processed and visualized using Cytogenomics 4.0.2.21 software (Agilent, Santa Clara, CA, USA).

### 2.10. Immunofluorescence Studies

For the RPE characterization studies, iPSC-derived RPE was seeded and processed as previously described (19). For the differentiation assay, embryoid bodies were fixed with 4% PFA, permeabilized with 0.1% Triton X-100 and blocked in 10% donkey serum (Millipore) and 1% BSA (Sigma-Aldrich). Primary antibodies were diluted in 1% donkey serum and 1% BSA, and incubated overnight at 4 °C. Secondary antibodies were incubated 45 min at room temperature with 0.2 µg/ml bisBenzimide Hoechst (Sigma-Aldrich) prior to mounting in ProLong Diamond Antifade Mountant (Molecular probes, Life Technologies). Primary antibodies: 1:100 dilution rabbit anti-human ZO-1 (Invitrogen, Life technologies), 1:250 rabbit anti-human LRAT (Abcam), 1:500 mouse anti-human Bestrophin-1 (Abcam), 1:200 mouse anti-Nestin (Novus Biologicals, Lille, France), 1:200 mouse anti-SMA (Dako, Les Ulis, France) and 1:200 mouse anti-AFP (Sigma-Aldrich). Secondary antibodies: 1:500 dilution donkey anti-rabbit IgG-Alexa Fluor 594, and donkey anti-mouse IgG-Alexa Fluor 488 (Jackson ImmunoResearch). Images were taken using a Zeiss ApoTome 2 Upright wide-field microscope (Carl Zeiss SAS).

### 2.11. Transmission Electron Microscopy

Eight-weeks post-passaging, the iPSC-derived RPE culture inserts were processed and embedded as previously described without modification [19]. Thin (70 nm) sections were counterstained and observed using a Tecnai F20 transmission electron microscope at 200KV (CoMET, MRI facility).

### 2.12. Transepithelial Resistance Measurements

The TER of the iPSC-derived RPE culture inserts was measured using the Epithelial Volt/Ohm Meter EVOM2 (World Precision Instruments, Hertfordshire, UK) as described [19]. The background value of a cell-free Matrigel-coated insert was subtracted from the recording and the value multiplied by the growth surface area. Final TER values, expressed as Ω/cm^2^, represent the average measure of three filters.

### 2.13. Phagocytosis Assay

Dissected and homogenized bovine neuroretinas were placed in 20% sucrose and loaded onto a discontinuous 20–60% sucrose gradient. The gradient was ultracentrifuged at 75,600× *g* for 1 h at 10 °C and the photoreceptor outer segments (POS) collected from the 40% layer. The POS were washed twice with HBSS (ThermoFischer Scientific), resuspended in 2.5% sucrose and the concentration determined by flow cytometry (BD Accuri flow cytometer, BD Biosciences). For the fluorescent labeling, the POS were washed in 0.1 M NaHCO_3_, pH = 9, centrifuged at 21,130× *g* for 10 min at RT and resuspended in 0.1 M NaHCO_3_ containing 1:10 dilution of FITC Isomer I (Invitrogen). The samples were incubated overnight at 4 °C in the dark, then washed in PBS and resuspended in ES media. For the phagocytosis assay, two 0.32 cm^2^ wells of iPSC-derived RPE per condition were incubated with 10 POS/cell for 2.5 h at 37 °C, washed, dissociated and analyzed by flow cytometry.

### 2.14. In Vitro Prenylation Assay

iPSC-derived RPE grown in 1.9 cm^2^ wells (one well per condition) was collected and resuspended in cold, freshly prepared, degased prenylation lysis buffer as previously described [13]. The freshly prepared lysate was incubated with 9 µM biotin-labelled geranyl pyrophosphate (B-GPP; Euromedex, Souffelweyersheim, France), 22 µM GDP, 70 ng/µL recombinant REP1 (Euromedex) and 80 ng/µL recombinant RGGTase II (Euromedex) at 37 °C for 1 h, and the unprenylated cytosolic Rab pool was analyzed by western blot analysis as described without modification [19]. The amount of biotinylated Rab proteins was quantified using Image J software and expressed as a function of the β-actin signal.

### 2.15. Statistical Analysis

Statistical analyses of three or more groups were performed using a Kruskal Wallis test and post-hoc 2 × 2 comparisons using a Mann and Whitney test.

## 3. Results

### 3.1. Typical Choroideremia Clinical Phenotype

**Figure 1:**
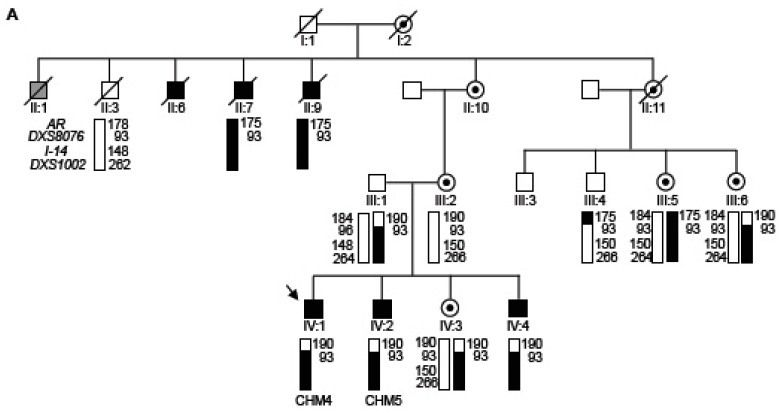

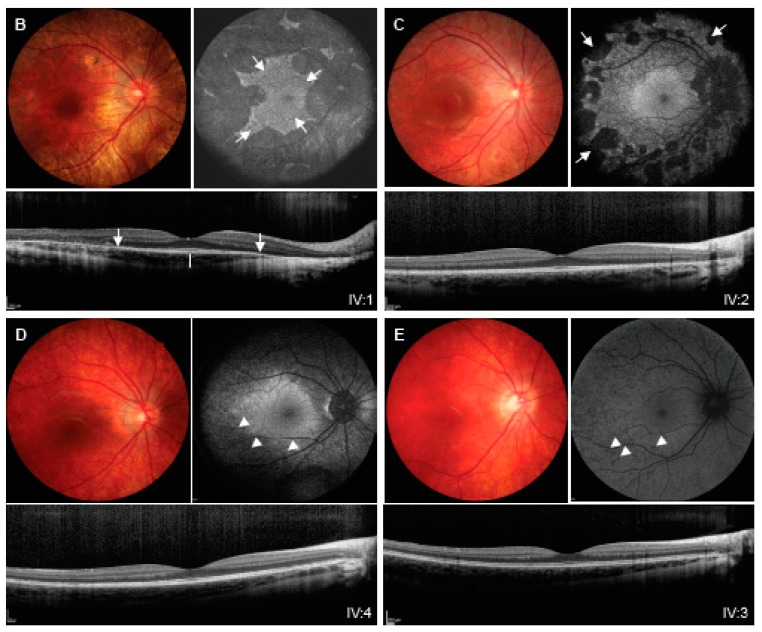
Family history and clinical phenotype. (**A**) Pedigree indicating males affected with choroideremia (filled symbols), carrier females (symbols with a dark dot) and unaffected individuals (open symbols). Diagonal lines indicate deceased individuals. In generation II, grey symbol indicates an individual who was reported as blind due to diabetes mellitus. The numbering of individuals in this generation is not continuous, as four additional siblings who died in childhood have not been indicated on the pedigree. The proband, (individual IV:1; CHM4) is indicated by an arrow. Disease-associated haplotypes are indicated by black rectangles and normal haplotypes by white rectangles. A recombination between the normal and *CHM* allele (black and white rectangles) appears to have occurred. AR—polymorphic marker in the gene encoding the androgen receptor. DXS8076 and DSX1002—polymorphic markers flanking the *CHM* gene. I-14—single tandem repeat in intron 14 of *CHM.* (**B**) Multimodal imaging of the right eye of the 18 year-old proband IV:1. Upper left panel, fundus photographs showing the scalloped chorioretinal atrophy of the mid-periphery and preserved appearance of the posterior pole. Upper right panel, fundus autofluorescence highlighting the string-like pattern of the preserved macular area (white arrows) that contrasts with the dark appearance of the peripheral chorioretinal atrophy. Lower panel, SD-OCT scan showing a preserved ellipsoid zone at the posterior pole that is delimited by the two arrows. On the peripheral part of the scan, the ellipsoid zone vanishes and the outer nuclear layer is thinner. The white bar indicates the thinning of the choroid beneath the fovea. (**C**) The younger brother (VI:2; CHM5) at 14 years of age. Upper left panel, funduscopy showing a salt and pepper pattern of the mid-periphery of the retina. By autofluorescence imaging (upper right panel), the atrophic patches are better identified as they appear black (white arrows) and contrast with the preserved posterior pole. By SD-OCT, the ellipsoid zone is identifiable on the entire scan. (**D**) The youngest brother (IV:4) at 9 years of age with early stage lesions. Upper left panel, typical multiple mottling of the retinal pigment epithelium can be seen in the mid-periphery of the fundus. Upper right panel, autofluorescence imaging discloses multiple small hypo-autofluorescent dots (arrowheads) but no complete atrophic lesions. The posterior pole is preserved and has an almost normal appearance on the SD OCT scan (lower panel). (**E**) The asymptomatic heterozygous sister (IV:3) with a typical reticular pattern of pigmentary mottling of the retinal pigment epithelium (upper left panel) and hypo-autofluorescent thin dots or speckles (arrowheads) in the mid-periphery and outer part of the posterior pole (upper right panel). There are no significant lesions detectable on the SD-OCT scan (lower panel).

The CHM family reported herein comprises four siblings (three affected males and one carrier female) born from a female carrier (individual III:1; Figure 1A). The proband in this study is individual IV:1, herein referred to as CHM4 in reference to an anonymized chronological ordering of our patient fibroblast bank. The segregating *CHM* mutation is a previously reported 1.52 Mb deletion at Xq21.2, which removes the entire gene [29,32]. Haplotype analysis of the extended family suggested that the deletion segregated from the maternal great grandmother to all four siblings (Figure 1A).

At the clinical level, the proband was examined at 18 years of age. Visual acuity was 20/20 in both eyes with −0.75 (−1.25, 45°) in the right eye and (−1.00, 155°) in the left eye. Fundus examination showed peripheral patches of atrophy of the choroid and the RPE with a large preserved macular area (Figure 1B). On a Goldmann visual field, the peripheral isopter V4 was preserved with several small annular scotoma in the 40–60° area (data not shown). By SD-OCT, the outer retina was preserved and well segmented in the foveal zone (Figure 1B). The ellipsoid zone and the outer nuclear layer were absent in areas with atrophy of the choroid and the outer retina. Fundus autofluorescence detected a large preserved posterior pole surrounded by hypoautofluorescent patches of RPE and choroid atrophy (Figure 1B). 

The two younger brothers of the proband were asymptomatic at 14 years (individual IV:2; herein referred to as CHM5) and 9 years (individual IV:4) of age, with a visual acuity of 20/20 in both eyes. Goldmann visual field was unremarkable (data not shown). Individual IV:2 (CHM5) had a few mid-peripheral patches of chorioretinal atrophy (Figure 1C) and individual IV:4 had patchy pigment mottling of the peripheral retina (Figure 1D). SD-OCT did not detect major alterations in either brother. The full-field electroretinogram responses of the three affected brothers were not recordable in dark-adapted conditions whereas discernible but reduced responses were detected in light-adapted conditions (data not shown). The proband’s sister (individual IV:3) is a CHM carrier with patchy pigment mottling and areas of hypopigmentation in both eyes (Figure 1E), as was also the case for the mother (data not shown). Fundus autofluorescence demonstrated multiple small, round hypoautofluorescent lesions at the posterior pole and in the mid-peripheral retina (Figure 1E).

Taken together, the clinical profiles of the family members are typical of CHM and consistent with the X-linked segregation pattern.

### 3.2. Lack of REP1 Production in the iPSCs of CHM4 and CHM5

**Figure 2:**
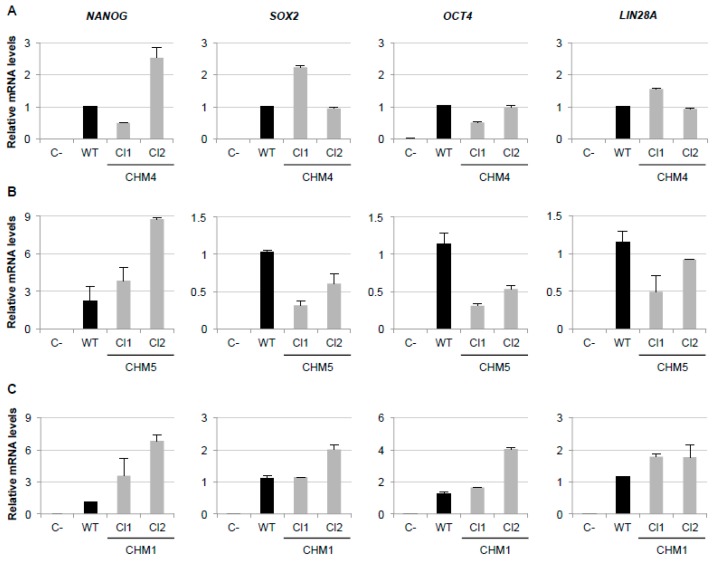

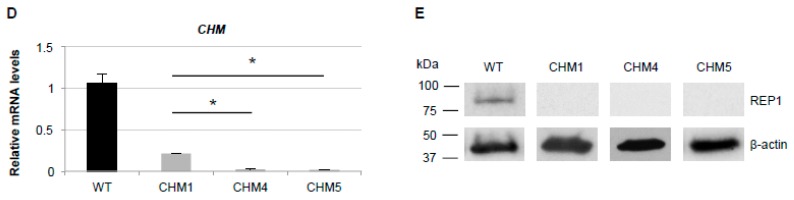
Pluripotency and *CHM* expression of the generated iPSCs. qPCR analysis showing an expression of endogenous *NANOG, SOX2, OCT4* and *LIN28A*, relative to *GAPDH* expression, in CHM4 (**A**), CHM5 (**B**) and CHM1 (**C**) iPSCs (grey bars), as compared to the absence of expression in fibroblasts (C-). As a positive control, the same pluripotency markers were expressed in a previously validated wild type iPSC line (black bars). Cl1 and Cl2 indicate clone 1 and clone 2, respectively. (**D**) qPCR analysis of *CHM* expression (relative to *GAPDH*) shows that the *CHM* deletion carried by the CHM4 and CHM5 iPSCs results in the absence of a *CHM* transcript (one clone shown for each cell line). This was significantly different to the residual transcript levels in the CHM1 iPSCs, which carry a truncating frameshift mutation (data expressed as mean ± SEM, *n*=3; Mann and Whitney test *p* < 0.05). (**E**) REP1 protein was not detected in the CHM1, CHM4 and CHM5 iPSCs (one clone shown for each cell line), as compared to wild type (WT) cells. β-actin expression serves as the loading control.

In 2014, we reported for the first time the generation of iPSCs for choroideremia, from a patient referred to as CHM1, using a lentivirus vector platform [13]. Although we obtained genetically stable iPSCs for CHM1, reprogramming efficiency was low. We thus switched to the non-integrative Sendai virus platform and obtained genetically stable iPSCs for two other patients (CHM3 and CHM6) with a notable increase in efficiency [19,20]. Similarly here, we used Sendai virus vectors to successfully reprogram the fibroblasts from the proband (IV:1; CHM4) and his brother (IV:2; CHM5). We selected and amplified two iPSC clones for each line. We assayed for pluripotency by qPCR analysis of the expression of the endogenous markers *NANOG, SOX2, OCT4* and *LIN28A*. Both the CHM4 (Figure 2A) and CHM5 (Figure 2B) iPSC lines expressed the pluripotency markers similar to a previously validated wild type iPSC line. By contrast, no expression could be detected in fibroblast controls (C-). In parallel, to homogenize the origin of our CHM iPSC bank, we reprogrammed the fibroblasts of CHM1 using the non-integrative Sendai vector method and determined the pluripotency of two selected clones by qPCR analysis. Both clones expressed the pluripotency markers similar to the wild type control (Figure 2C). We next tested *CHM* transcript levels in control and patient iPSCs by qPCR analysis. No *CHM* expression was detected in the iPSCs of patients CHM4 and CHM5 in comparison with the reduced expression (20% of wild type) detected for the CHM1 cells (Figure 2D). Consistently, REP1 expression was not detected in the iPSCs of patients CHM4 or CHM5 by western blot analysis (Figure 2E), compared to wild type cells. Similarly, REP1 expression was not detected in the CHM1 iPSCs consistent with our previous observations [13].

Therefore, the pluripotent iPSCs generated from for CHM4 and CHM5 did not show detectable *CHM* transcript or REP1 levels consistent with the deletion of the *CHM* gene.

### 3.3. High Level of Genetic Instability in the iPSCs of CHM4 and CHM5

**Figure 3:**
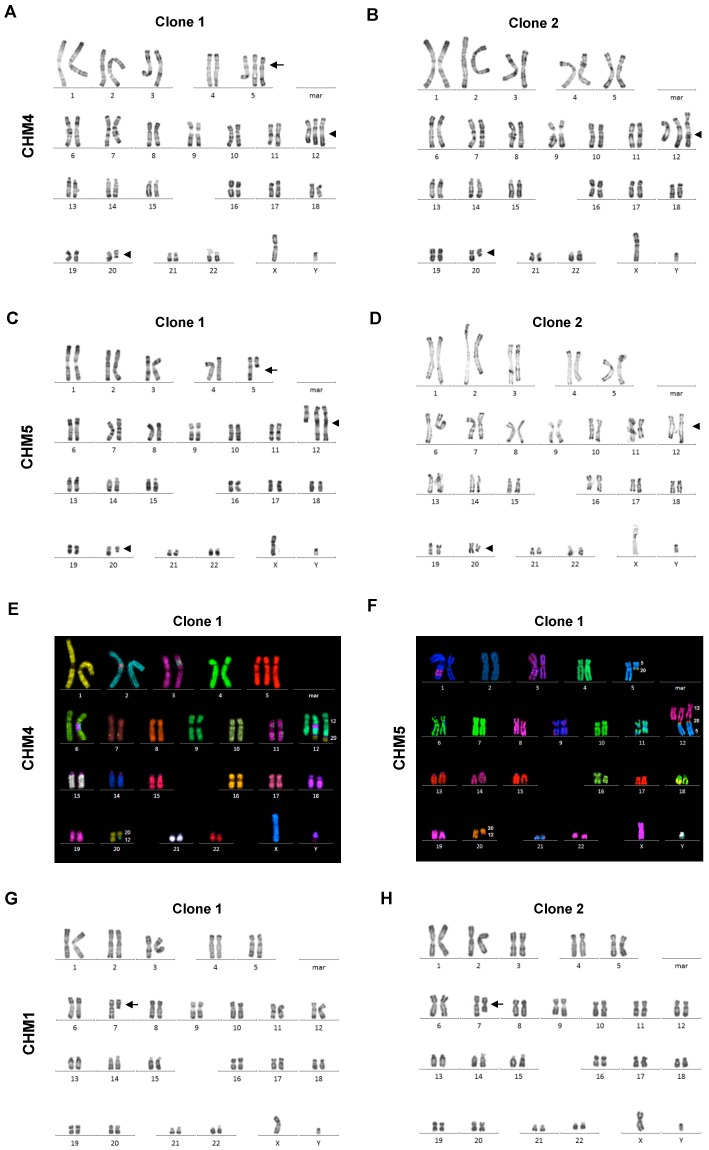
Genetic instability of the generated iPSCs. (**A**) RHG banding of clone 1 of CHM4 detected a trisomy of chromosome 5 (arrow) and translocation between chromosome 12 and chromosome 20 (arrowheads). The rearranged chromosome 12 was duplicated. (**B**) RHG banding of clone 2 detected the same rearrangements of chromosomes 12 and 20, including a duplication of the derivative chromosome 12 (arrowheads), whereas no rearrangement of chromosome 5 could be detected. (**C**) RHG banding of clone 1 of CHM5 detected a loss of the long arm of chromosome 5 (arrow) and the same translocation between chromosome 12 and chromosome 20 (arrowheads) detected in (A). (**D**) RHG banding of clone 2 exclusively detected the translocation between chromosomes 12 and 20 (arrowheads). (**E**) Multicolor FISH of the clone 1 of CHM4 shows the trisomy 5 in red, a normal chromosome 12 in green, a normal chromosome 20 in yellow and the derivative 12;20 chromosomes in green and yellow. (**F**) Multicolor FISH of clone 1 of CHM 5 shows that part of chromosome 20 (in orange) is also added to the truncated chromosome 5 (in blue) and that the majority of the long arm of chromosome 5 is added to the derivative chromosome 12 (in pink, orange and blue), which is duplicated. The derivative chromosome 20 can be seen in orange and blue. (**G**) Karyotype analysis by RHG banding of clone 1 of CHM1 detected a deletion of the long arm of chromosome 7 (arrow). (**H**) In clone 2 the long arm of chromosome 7 was deleted and replaced by a duplication of the short arm (arrow).

The iPSC clones for CHM4 and CHM5 were then amplified for karyotype analysis. Unexpectedly, both cell lines showed the presence of chromosomal aberrations (summarized in Table 1). The karyotype anomalies were complex and involved combinations of the chromosomes 12, 20 and/or 5. In both clones of the cell line CHM4, we detected a translocation between chromosome 12 and 20, which we denoted t(12;20)(q24.3;q11.2), and the derivative chromosome 12 was also duplicated (Figure 3A,B). In addition, in clone 1, a trisomy of chromosome 5 was also observed (Figure 3A). In the cell line CHM5, we detected the t(12;20)(q24.3;q11.2) translocation in both clones (Figure 3C,D). Furthermore, in clone 1, this translocation was also associated with a rearrangement of chromosome 5 (Figure 3C). Multicolor FISH labeling was used to more clearly delineate the more complex rearrangements. In clone 1 of CHM4, the trisomy of chromosome 5, the translocation between chromosomes 12 and 20, and the duplication of the derivative chromosome 12 was confirmed (Figure 3E). In clone 1 of CHM5, the long arm of chromosome 5 was added to the derivative chromosome 12, which was duplicated. Moreover, part of chromosome 20 was added distal to the centromere of the aberrant chromosome 5 (Figure 3F). By contrast, by karyotype analysis we detected relatively simple anomalies in the clones of CHM1, which involved the loss of the long arm of chromosome 7 distal to q21 in clone 1 (Figure 3G). In clone 2, an isochromosome 7p was detected, whereby the entire long arm of chromosome 7 was lost and the short arm was duplicated (Figure 3H). 

Taken together, the iPSCs for CHM4 and CHM5 were genetically unstable with complex aberrations involving chromosomes 12, 20 and/or 5, depending on the clone.

### 3.4. Familial Segregation of a Novel Chromosomal Translocation

**Figure 4:**
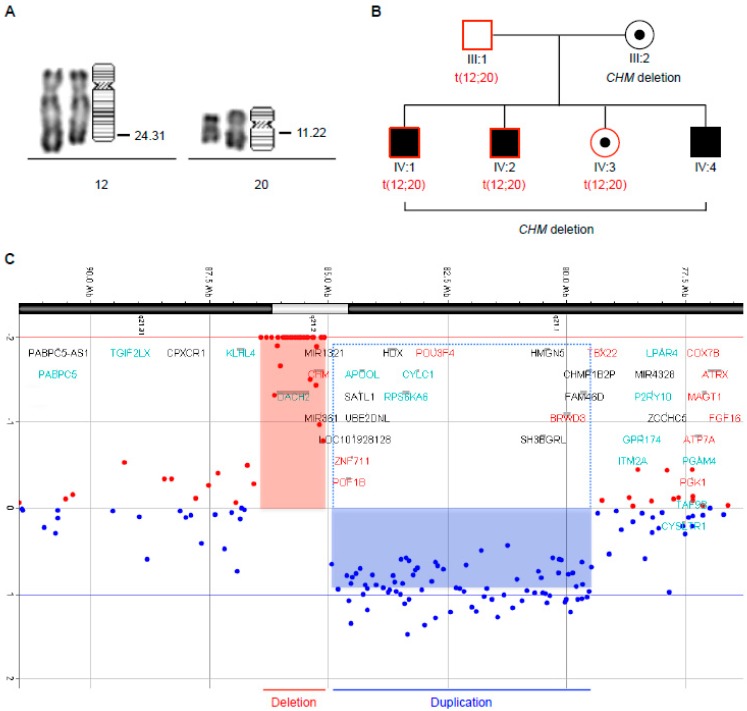
Analysis and segregation of chromosomal anomalies. (**A**) Partial karyotype by RHG banding of the fibroblasts of individual CHM5 and ideogram showing the breakpoints of the reciprocal translocation t(12;20)(q24.3;q11.2). (**B**) Results of blood karyotyping of all the individuals from the immediate family of the proband, generations III and IV of the pedigree shown in Figure 1 demonstrated that the translocation t(12;20)(q24.3;q11.2) (indicated as t(12;20)) was transmitted from the father to all the siblings (red outlines) with the exception of the youngest brother. The complementary segregation of the *CHM* deletion from the mother to all the siblings is also indicated (black shading or black dot). (**C**) aCGH profile of chromosome X of individual CHM5 showing chromosome copy number (Y axis; log_2_ ratio) and the chromosome region they span (X axis). The 1.5-Mb deletion (red box) spans the genes *CHM* and *DACH2*. The 5.4-Mb duplication (blue box) spans the genes *FAM46D*, *BRWD3*, *HMGN5*, *SH3BGRL*, *POU3F4*, *CYLC1*, *RPS6KA6*, *HDX*, *APOOL*, *SATL1*, *ZNF711*, and *POF1B* (outlined by dashed box).

As we had previously generated genetically stable iPSCs with the fibroblasts of patient CHM1, we knew that the anomalies in the Sendai vector-generated CHM1 iPSCs arose post-reprogramming. In order to determine if this was also the case with the CHM4 and CHM5 iPSCs, we performed karyotype analysis of the original fibroblasts. We detected the t(12;20)(q24.3;q11.2) translocation (Figure 4A) but no other rearrangements in the cells of both patients. To rule out a tissue culture artifact, we extracted blood from all six members of the family, individuals III:1, III:2, IV:1, IV:2, IV:3 and IV:4, and performed karyotype analysis. We detected the translocation in the father (individual III:1) and in three of the four children: individual IV:1 (CHM4), individual IV:2 (CHM5) and their sister, individual IV:3 (schematically summarized in Figure 4B). By contrast, the youngest son (individual IV:4) did not inherit the rearrangement. In comparison, the maternally inherited *CHM* deletion was transmitted to all four siblings. Taken together, of the iPSCs generated, only clone 2 of CHM5, which exclusively carried the t(12;20)(q24.3;q11.2) translocation, represented a genetically stable iPSC line.

To determine if the t(12;20)(q24.3;q11.2) translocation resulted in a gain or loss of genetic material in the patients, we performed an aCGH analysis of the fibroblasts of CHM4 and CHM5. At the resolution of the microarray used, we did not detect any copy number variations at the chromosomal regions 12q24.3 and 20q11.2, suggesting that the translocation was balanced. Furthermore, we confirmed the presence of the causative *CHM* deletion at Xq21, arr(GRCh37/hg19) Xq21.2-q21.31(85089328-86428594)x0, which wholly encompassed the genes *CHM* (85116185-85302566) and *DACH2* (85403462-86087607). However, in addition, we detected a previously unreported 5.4 Mb duplication of the region Xq21.1-q21.2(79502972-84948148)x2 associated with the causative *CHM* deletion, which encompassed the genes *FAM46D*, *BRWD3*, *HMGN5*, *SH3BGRL*, *POU3F4*, *CYLC1*, *RPS6KA6*, *HDX*, *APOOL*, *SATL1*, *ZNF711*, and *POF1B* (Figure 4C). 

To conclude, we identified a novel reciprocal translocation, t(12;20)(q24.3;q11.2), with a paternal segregation in this family. Moreover, we determined that the maternally inherited causative *CHM* deletion was associated with a 5.4-Mb duplication.

### 3.5. CHM Retinal Phenotype Is Not Further Impacted by the Translocation or Duplication

**Figure 5:**
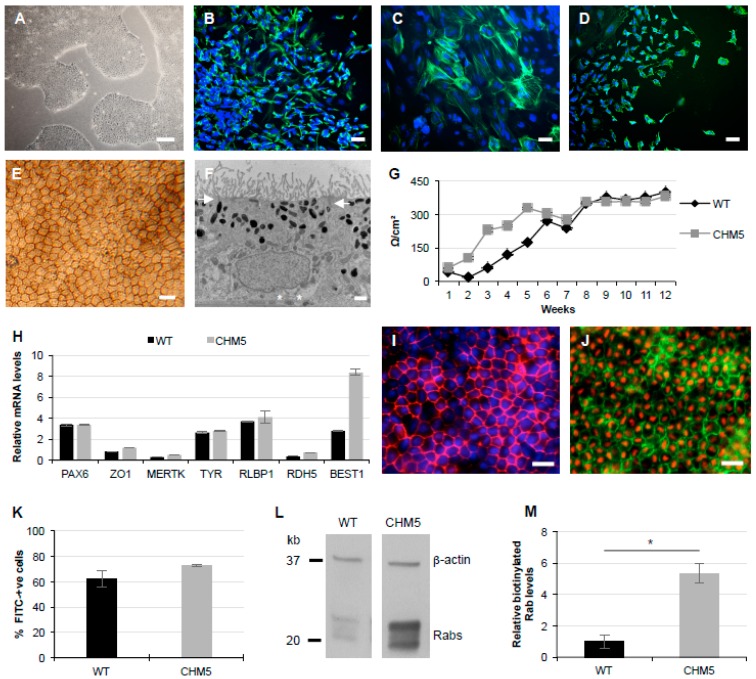
Characterization of CHM5 iPSC-derived RPE. (**A**) Bright-field microscopy of CHM5 iPSC colonies cultured under feeder-free conditions. Scale bar = 200 µm. Embryoid bodies staining positive for the ectoderm marker Nestin (**B**), the mesoderm marker SMA (**C**) and the endoderm marker AFP (**D**). Scale bars = 50 µm. (**E**) Bright-field microscopy of the pigmented and cobblestoned iPSC-derived RPE monolayer. Scale bar = 20 µm. (**F**) Transmission electron micrograph of the polarized iPSC-derived monolayer showing an RPE cell with apical microvilli, apical tight junctions (arrows), apically located melanosomes, basally located nucleus and basally secreted collagen (asterisks). Scale bar = 1 µm. (**G**) TER measurements in normalized Ω/cm^2^ of the CMH5 iPSC-derived RPE (grey curve) as a function of the number of weeks after P3 seeding in comparison to the wild type (WT; black curve). Data expressed as mean ± SEM, *n* = 3. (**H**) qPCR analysis of the expression in relative units of typical RPE genes in the CHM5 iPSC-derived RPE in comparison to wild type (WT). Data normalized to *GAPDH* expression and expressed as mean ± SEM, *n* = 3. (**I**) IF studies of the CHM5 iPSC-derived RPE showing expression of apical ZO1 (in red; nuclei labelled in blue) Scale bar = 20 µm. (**J**) IF studies of the CHM5 iPSC-derived RPE showing expression of baso-lateral Bestrophin-1 (in green) and peri-nuclear LRAT (in red). Scale bar = 20 µm. (**K**) Phagocytosis assay performed in duplicate of the percentage of CHM5 iPSC-derived RPE cells (grey bar) with internalized FITC-labelled bovine POS in comparison to the wild type (WT; black bar). Data expressed as mean ± SEM. (**L**) Western blot showing the larger biotinylated Rab pool in CHM5 iPSC-derived RPE in comparison to the wild type (WT). β-actin expression represents the loading control. (**M**) Semi-quantification of two independent prenylation assays showing a significant 5-fold higher level of biotinylated Rabs in CHM5 iPSC-derived RPE compared to the wild type (WT). Data expressed as mean ± SEM; Mann and Whitney test *p* < 0.05).

We previously showed that iPSC-derived RPE from CHM patients reproduces the biochemical defect of the disease, i.e., an underprenylation of Rab GTPases due to defective or absent REP1 expression [13,19,20]. Thus, to assay the retinal phenotype in this family, we generated iPSC-derived RPE from clone 2 of CHM5. To this end, we first used an embryoid body assay to determine whether this clone (Figure 5A) could differentiate into the three germ layers. Immunofluorescence (IF) studies of the CHM5 embryoid bodies identified ectoderm layers, as determined by Nestin expression (Figure 5B), mesoderm layers by Smooth Muscle Actin (SMA) expression (Figure 5C) and endoderm layers by α-fetoprotein (AFP) expression (Figure 5D). 

We then differentiated the iPSCs into RPE, which, from P3, consisted of a monolayer of polygonal pigmented cells (Figure 5E). Transmission electron microscopy studies showed a polarized monolayer with apical microvilli and tight junctions between cells, apically located melanosomes accounting for the pigmentation, basal nuclei, and basally secreted collagen (Figure 5F). Consistent with the expression of ZO1, the RPE was confirmed as tight (>150 Ω/cm^2^) by measuring the transepithelial resistance (TER) of the tissue. The TER of both wild type and CHM5 RPE increased until 8 weeks post-seeding whereupon it remained stable (Figure 5G). Furthermore, qPCR analysis showed expression of typical RPE markers in the CHM5 iPSC-derived RPE at levels similar to or superior to wild type: *PAX6*, an early differentiation marker; *ZO1* and *MERTK*, apical tight junction and microvilli markers, respectively; *TYR*, a melanosome marker; *RLBP1* and *RDH5*, cytosolic visual cycle markers, and *BEST1*, a baso-lateral membrane marker (Figure 5H). We also used IF studies to confirm the expression of the RPE-specific markers ZO1 (Figure 5I) and Bestrophin-I (Figure 5J), as well as the visual cycle protein LRAT (Figure 5J). Furthermore, the iPSC-derived RPE was functional as determined by its ability to phagocytize FITC-labelled photoreceptor outer segments (POS) at levels similar to wild type RPE, as determined by flow cytometry (Figure 5K).

Lastly, we assayed for a prenylation defect in CHM5 iPSC-derived RPE in comparison to wild type cells using an in vitro prenylation assay. In this assay, the cytosolic cell fraction is isolated and a biotinylated prenyl donor is added. If an unprenylated Rab GTPase population is available in the cytosol, the newly prenylated Rabs will be detectable by western blot analysis, and the intensity of the signal will be proportional to the size of the unprenylated pool. In wild type iPSC-derived RPE a faint biotin signal is detected following western blot analysis in contrast to the stronger signal seen in the CHM5 RPE (Figure 5L). A semi-quantification of two independent assays showed a significant 5-fold increase in cytosolic Rab content in CHM5 iPSC-derived RPE compared to the wild type (Figure 5M). This fold difference is in the range of our previous observations on iPSC-derived RPE carrying *CHM* mutations [13,19,20].

Taken together, the iPSCs of patient CHM5 differentiated into *bona fide* RPE that showed an underprenylation defect comparable to that of other patients.

## 4. Discussion

Stem cell technology has revolutionized the study of inherited diseases in terms of validating causative genes, identifying disease pathways, and testing the efficiency of novel therapeutics. The generation of iPSCs, which originate from somatic cells, has further facilitated this research axis as it avoids ethical considerations associated with the use of human embryonic stem cells. However, the genetic instability associated with iPSC generation calls for rigorous quality controls. Over recent years, we have generated a bank of fibroblasts and genetically stable iPSCs from individuals affected with the IRD CHM for both mutation validation [29,33] and therapeutic [13,19,20] studies. Here, we describe a novel CHM family carrying a deletion of the entire *CHM* gene. Intriguingly, and in contrast with our past experience, karyotype analysis of the iPSCs generated from two brothers of this family (CHM4 and CHM5) showed a high level of genetic instability. Underlying this instability, we identified a novel reciprocal chromosomal translocation, t(12;20)(q24.3;q11.2), that was segregating in the family. Thus, we show for the first time, that an existing instability in somatic cells can exacerbate the genetic instability associated with iPSCs. 

The CHM phenotype in the family results from a maternally inherited 1.52-Mb deletion at Xq21.2-21.3 on the X chromosome [29,32] that removes the entire *CHM* gene and presumably the promoter region [34]. This correlates with the absence of a *CHM* transcript and REP1 in the cells of individuals CHM4 and CHM5. *DACH2* expression was not directly tested in these patients but, as the deletion removes the 5′ part of the gene, it is likely that it abolishes gene expression. However, it is unlikely that the *DACH2* deletion impacts the phenotype, as studies in the mouse *Dach2^-/-^* model suggest that there is a compensation of its function by another member of the same family, *Dach1* [35] (murine homologue of human *DACH1*; MIM 603803). Moreover, in cases where *DACH2* variants have been identified in individuals with disease phenotypes, variants in a second gene were also identified [36,37]. Therefore, to date, there is no conclusive evidence linking *DACH2* to a clinical phenotype.

We further determined that the causative *CHM* deletion is associated with a previously unreported 5.4-Mb duplication. There are twelve genes located within this duplication, half of which have been linked to a clinical phenotype. Although the exact function of the zinc finger protein *ZNF711* is unknown, variants in this gene have been described as associated with cognitive disability [38]. Similarly, mutations in *BRWD3*, which may have a chromatin-binding function, have been associated with cognitive disabilities [39,40], and the ribosomal S6-kinase, *RPS6KA6*, is commonly deleted in patients with X-linked mental retardation [41]. Disruption of *HDX,* encoding a highly divergent homeobox protein with DNA-binding activity [42], and *POF1B*, encoding an actin-binding protein [43], have both been associated with premature ovarian failure. Mutations in *POU3F4* have been associated with X-linked deafness type 3 [44,45]. Interestingly, there has been a report of a female patient carrying a balanced chromosomal translocation t(X;4)(q21.2;p16.3) and presenting with CHM, mild sensorineural deafness and primary ovarian failure [46,47]. Of the remaining genes, the function of *FAM46D* and *SH3BGRL* is currently unknown, and *HMGN5*, *CYLC1*, *APOOL* and *SATL1* (paralogue of *SAT1*), although their roles have been elucidated, have not been linked to a clinical phenotype. To our knowledge, the effect of a potential duplication of these genes is unknown with the exception of *APOOL*. *APOOL* encodes a cardiolipin-binding protein, which, when overexpressed, leads to deregulation of mitochondrial cristae morphology [48]. In males it has been reported that large duplications of the X chromosome can be benign [49]. Nonetheless, the multiple rearrangements present in this family argue for a more extensive extra-ocular clinical evaluation, with particular focus on neurological testing.

The segregating t(12;20)(q24.3;q11.2) translocation identified in this family does not appear to negatively impact the health of the individuals, as it is carried by the unaffected father. This is consistent with the aCGH array data that suggest that this translocation is balanced. Generally, balanced reciprocal translocations do not have a phenotypic effect in carriers, but they can lead to meiotic instability and thus give rise to reproductive problems, usually recurrent pregnancy loss, chromosomally abnormal offspring or infertility [50,51,52], as well as to full-term pregnancies with birth defects [53]. Parental chromosomal rearrangements have been reported in 2–5% of couples with recurrent miscarriages. However, the reproductive risks may be different for male and female carriers of the same translocation. One report of a paternally inherited translocation resulted in four miscarriages in the carrier daughter [54]. Therefore, although the t(12;20)(q24.3;q11.2) translocation did not appear to provoke infertility in the father, or recurrent miscarriages within the couple, the situation may be different for his offspring, in particular his daughter. Furthermore, the susceptibility of the iPSCs carrying the reciprocal translocation to undergo further extensive rearrangements, as shown in this study, may be indicative of the imbalances that could occur in an embryo. It is now established that an embryo from a carrier of a balanced chromosomal abnormality exhibits more instability and shows an increased rate of chromosomal mis-segregation leading to a higher number of aneuploidies than a normal embryo [55]. The presence of an abnormal chromosome, such as a derivative chromosome from a Robertsonian translocation, destabilizes the mitotic spindle symmetry leading to an abnormal segregation of the structurally normal chromosome. This phenomenon, known as an inter-chromosomal effect [56], can also explain the exacerbation of genetic instability associated with iPSCs.

Concerning carriers of chromosomal abnormalities, an *in silico* analysis with the database HC forum (www.hc-forum.net) [57] predicted that the most likely modes of segregation for the t(12;20)(q24.3;q11.2) rearrangement in the offspring are adjacent 1 (whereby a rearranged chromosome is transmitted with a normal homologue) and 3:1 (whereby a rearranged chromosome is transmitted with its homologue and with a normal homologue, or with two normal homologues). These segregation modes will result in the potential formation of viable unbalanced zygotes carrying trisomy of the normal, or of the derivative, chromosomes 12 or 20. This predictive meiosis segregation is reminiscent of our results showing a duplication of the derivative chromosome 12 in one of the clones of both patients. Furthermore, HC forum predicted a risk of approximately 20% of a viable birth with poly-malformative syndrome and/or intellectual disabilities. Consequently, the three siblings should be advised to undergo genetic counseling when family planning is envisaged.

Despite their significant advantages, iPSCs do harbor some limitations. In vivo, the renewal potential of PSCs is only transient but, by contrast, in vitro, this characteristic is exploited indefinitely and hence is associated with the risk of the occurrence of genetic instability [27]. PSCs have shorter cell cycles than differentiated cells due to a shortened G1 phase [58]. Therefore, when somatic cells are reprogrammed into iPSCs, they begin to proliferate rapidly and acquire short cell cycles, which incur a higher risk for the acquisition of genetic aberrations. Furthermore, this increase in successive rounds of DNA replication imposes a major hurdle for the DNA machinery, thus allowing replication defects and defective chromosomal segregation to pass through weakened checkpoints [27]. It has been estimated that ~20% of human iPSC lines generated exhibit at least one large chromosomal aberration [27]. Furthermore, recurrent aberrations have been identified and these most commonly involve gains of chromosomes 1, 12, 17 and 20, or fragments thereof [59]. In general, it is thought that these genetic changes provide variant cells with a growth advantage.

Interestingly, the t(12;20)(q24.3;q11.2) rearrangement reported herein involves two of the four chromosomes that are sites of recurrent aberrations in iPSCs. More precisely, a recurrent genomic instability of 20q11.21 has been identified in PSCs, which, when amplified, provides a selective advantage [59,60,61]. Along this line, the amplification of this region is associated with a variety of cancers [60]. Similarly, partial or full trisomies of chromosome 12, notably 12p, have also been associated with iPSCs [62,63]. These gains were linked to an overexpression of the pluripotency genes *NANOG* and *GDF3* and cell cycle genes, thus likely conferring growth advantage. The complex aberrations that we report here in the iPSC clones CHM4 and CHM5 that carry the t(12;20)(q24.3;q11.2) translocation, resulted in a partial trisomy of both chromosomes 12 and 20, and thus are also likely to have resulted in positive selection. Furthermore, although less common, trisomy of chromosome 5 has also been reported in iPSCs [59], thus may also confer a selective advantage.

In contrast, the iPSC clones of CHM1 that were generated from genetically stable somatic cells showed much simpler anomalies consisting of a monosomy of 7q and/or a trisomy of 7p. Gains or losses of chromosome 7, in particular the loss of 7p, have been previously reported in multiple cell lines [59]. The complexity of the anomalies in the CHM4 and CHM5 iPSCs, compared to CHM1, strongly suggest that they built upon the breakpoints of the existing translocation. We have shown that these aberrations vary between clones of the same individual suggesting that the existing translocation confers an additional fragility to the cells, which can be the source of further random instability at each passage. This information is particularly important in an era where iPSC-based transplants are being performed in humans, and reinforces the need for strict quality controls [64]. Thus, if iPSCs are to be produced from patient cells that are known to carry rearrangements, particular care needs to be taken with the reprogramming process and passaging, and karyotype analysis should be rigorously performed.

Along this line, to reprogram the iPSCs in this study, we switched from an inefficient lentiviral reprogramming method [13] to a highly efficient Sendai viral method, which resulted in a large number of clones simultaneously emerging and requiring passaging. Furthermore, during this period, we were using a time-consuming mechanical passaging protocol and culturing the iPSCs on feeder layers, which comprised irradiated human fibroblasts. Accordingly, fluctuations in temperature and CO_2_ concentrations, as well as in feeder cell quality, may have resulted in sub-optimal culture conditions [65] and exacerbated the appearance of the rearrangements. This likely explains the chromosome 7 rearrangements in the CHM1 clones generated here, as compared to the stable line that we generated previously. Technological evolutions in recent years that allow reprogramming under feeder-free conditions and passaging using rapid chemical dissociation protocols, have, in our experience [66,67,68,69], helped limit the emergence of genetically unstable cells.

We have previously shown that wild type iPSC-derived RPE is morphologically characteristic and functional, and can be used to study host-cell interactions for pathogens, such as Zika virus [70,71]. Moreover, we showed that choroideremia-specific iPSC-derived RPE mimics the biochemical defect of patients and is a highly pertinent model for proof-of-concept gene therapy studies [13,19] and for testing the efficiency of pharmacological agents [20]. Similarly, we differentiated the cells of patient CHM5 into iPSC-derived RPE and examined general RPE phenotype and function, as well as the CHM-specific biochemical defect. We show that the CHM5 RPE is morphologically and functionally characteristic of the wild type RPE. Furthermore, we show that the prenylation defect is similar to that measured in the fibroblasts of the same patient [29] as well as in the iPSC-derived RPE of other CHM patients with loss-of-function mutations [13,19,20]. Therefore, the cumulative chromosomal anomalies in this family do not appear to further impact the retinal phenotype. However, the family should be evaluated closely over time, as it cannot be excluded that the anomalies have an effect on other functions, notably fertility.

In conclusion, we present a novel outcome for iPSC technology whereby the high level of complex genetic instability resulted in the identification of a segregating translocation, which will need to be taken into account for future family planning. The important take-home message is that the detection of chromosomal aberrations in iPSC culture should be carefully analyzed, as, in addition to influencing the interpretation of biological studies, may reveal previously undetected rearrangements segregating in families.

## Figures and Tables

**Table 1:** Summary of the chromosomal aberrations carried by the CHM iPSC clones.

CHM iPSC Clones		Passage	Aberrations	Consequences
CHM4	Clone 1	P21	48,XY,+5,t(12;20)(q24.3;q11.2),+der(12)t(12;20)(q24.3;q11.2)	Trisomy 5, Partial trisomy 12, Partial trisomy 20
	Clone 2	P19	47,XY,t(12;20)(q24.3;q11.2), +der(12)t(12;20)	Partial trisomy 12, Partial trisomy 20
CHM5	Clone 1	P13	47,XY, der(5)(5pter ->q12::q13.1->qter),-12,+der(12)(12qter->q24.3::q11.2->q13.1::5q12->5qter)x2, der(20)(20qter->q11.2::12q24.3->12qter)	Partial trisomy 5, Partial trisomy 12, Partial trisomy 20
	Clone 2	P15	46,XY,t(12;20)(q24.3;q11.2)	Balanced
CHM1	Clone 1	P17	46,XY,del(7)(q21)	Monosomy distal to 7q21
	Clone 2	P19	46,XY,i(7)(p10)	Monosomy 7q, Trisomy 7p
